# Accumulation of Novel Amyloidosis‐associated proteins in the Progression of Alzheimer’s disease

**DOI:** 10.1002/alz.092565

**Published:** 2025-01-03

**Authors:** Wangchen Tsering, Kiara Lolo, Jennifer L Phillips, Todd Golde, Stefan Prokop

**Affiliations:** ^1^ University of Florida, Gainesville, FL USA; ^2^ Emory University, Atlanta, GA USA; ^3^ University of Florida / Center for Translational Research in Neurodegenerative Disease, Gainesville, FL USA

## Abstract

**Background:**

Alzheimer’s disease (AD) is one of the leading causes of death among seniors in the United States and costs the nation over $300 billion each year. Neuropathologically, AD is characterized by neuronal loss, Aβ deposits in the form of plaques, and intracellular aggregates of tau protein in the form of neurofibrillary tangles (NFT). The amyloid cascade hypothesis, one of the leading hypotheses of AD pathogenesis, suggests that Aβ aggregates are directly neurotoxic, triggering downstream neurodegeneration. However, direct evidence supporting the neurotoxicity of Aβ aggregates in vivo is lacking. For example, not all Aβ deposits elicit neurotoxicity. Diffuse plaques are typically negative for Congo‐Red and have very loose shapes and sizes, while neuritic plaques have congophilic cores and are surrounded by swollen neurites. If Aβ is directly neurotoxic, why does Aβ in diffuse plaques not elicit harmful responses while neuritic plaques are associated with neurodegenerative changes? Recent proteomic and transcriptomic studies suggest that there are many biologically active proteins that co‐accumulate with amyloid plaques, which are termed amyloidosis‐associated proteins (AAP). Most of AAP are signaling molecules, and some, such as APOE and Clusterin, are previously shown to be involved in AD pathophysiology. We evaluated the spatiotemporal distribution of diffuse plaques and neuritic plaques, and spatiotemporal accumulation of AAP in postmortem human AD brains of different AD neuropathological changes.

**Method:**

We used immunohistochemistry (IHC) and IF on post‐mortem brain tissue specimens to assess the disease stage and brain region specific distribution of NP and amyloidosis associated proteins in cases with low, intermediate and high AD neuropathological changes. In addition, we also use spatial biology to analyze protein level differences in different Aß plaque types.

**Result:**

We identified brain region and disease stage specific differences in the distribution and ratio of NP and other Aß‐deposits and correlated these findings with local microglia activation and co‐depositing proteins.

**Conclusion:**

Characterization and quantification of neuritic plaque and co‐depositing proteins in different brain regions and AD stages will guide effective therapeutic treatment for Alzheimer’s disease.

